# MSIreg: an R package for unsupervised coregistration of mass spectrometry and H&E images

**DOI:** 10.1093/bioinformatics/btae624

**Published:** 2024-10-17

**Authors:** Sai Srikanth Lakkimsetty, Andreas Weber, Kylie A Bemis, Verena Stehl, Peter Bronsert, Melanie C Föll, Olga Vitek

**Affiliations:** Khoury College of Computer Sciences, Northeastern University, Boston, MA 02115, United States; Institute of Surgical Pathology, University of Freiburg, Faculty of Medicine, Freiburg 79106, Germany; Faculty of Biology, University of Freiburg, Freiburg 79104, Germany; Khoury College of Computer Sciences, Northeastern University, Boston, MA 02115, United States; Institute of Pathology, Heinrich Heine University and University Hospital of Duesseldorf, Duesseldorf 40225, Germany; Institute of Surgical Pathology, University of Freiburg, Faculty of Medicine, Freiburg 79106, Germany; Tumorbank Comprehensive Cancer Center Freiburg, Medical Center, University of Freiburg, Freiburg 79106, Germany; Core Facility for Histopathology and Digital Pathology, Medical Center, University of Freiburg, Freiburg 79106, Germany; Khoury College of Computer Sciences, Northeastern University, Boston, MA 02115, United States; Institute of Surgical Pathology, University of Freiburg, Faculty of Medicine, Freiburg 79106, Germany; German Cancer Consortium and German Cancer Research Center, Heidelberg 69120, Germany; Khoury College of Computer Sciences, Northeastern University, Boston, MA 02115, United States

## Abstract

**Summary:**

Joint analysis of mass spectrometry images (MS images) and microscopy images of hematoxylin and eosin (H&E) stained tissues assists pathologists in characterizing the morphological structure of the tissues, and in performing diagnosis. Unfortunately, the analysis is undermined by substantial differences between these modalities in terms of aspect ratios, spatial resolution, number of channels in each image, as well as by large global or small local elastic spatial deformations of one image with respect to the other. Therefore, accurate coregistration of the images is a critical pre-requisite for their joint interpretation. We introduce MSIreg, an open-source R package for coregistration of MSI and H&E images. MSIreg is designed for high-dimensional MSI experiments where each spatial location is represented by thousands of mass features. Unlike most existing coregistration methods, MSIreg implements a landmark free workflow, and quantitative metrics for performance evaluation. We evaluate the performance of MSIreg on six case studies, including coregistration of contiguous tissues with large deformations, as well as simultaneous coregistration of 29 tissue microarray cores.

**Availability and implementation:**

The R package, installation instructions, and fully reproducible vignettes describing methods and Case Studies are available open-source under the GPL-3.0 license at https://github.com/sslakkimsetty/msireg/.

## 1 Introduction

Mass spectrometry imaging (MSI) is a powerful label free, high throughput technique that characterizes quantitative spatial distribution of hundreds of analytes ranging from small metabolites and lipids to peptides and proteins (Chen *et al.* 2023). MSI is being increasingly applied to biochemical analyses of diseases such as cancer (Kumar 2023), or to studies of drug metabolism and penetration (Tobias *et al.* 2019). Although MSI has “imaging” in its name, MS images are very different from natural images, generated, e.g. by microscopy. MSI experiments collect spectra with intensities of thousands of mass features at millions of tissue locations. A false color map of each mass feature across all the imaged locations forms one ion image. Therefore, MSI generates thousands of ion images for each tissue, each corresponding to a mass feature. Specialized software tools such as open-source Cardinal (Bemis *et al.* 2023) or commercial SCiLS Lab software (Bruker Daltonics, Bremen, Germany) are required to analyze these complex data.

While MSI characterizes the spatial distribution of biomolecules, histological microscopy images such as hematoxylin and eosin (H&E) stained tissue slides provide complementary morphological information. They help pathologists identify fine structures in the tissues, and perform diagnosis (Chan 2014). Therefore, it is advantageous to jointly analyze data from the two technologies, and interpret the molecular MSI information in a morphological context. Unfortunately, distortions arising from using different tissue sections for each modality or during tissue handling cause spatial misalignment of the data, and hamper direct image overlays.

Meaningful data integration is achieved by coregistering the two image modalities, i.e. by spatially transforming one image to align common and shared morphological features with the other.

General-purpose image coregistration methods are insufficient for MSI and H&E, for multiple reasons. MSI is highly multivariate with thousands of features in the spectral dimension and low spatial resolution, while microscopy has only up to four channels (including the alpha channel) and higher spatial resolution. Furthermore, the two modalities have different aspect ratios and intensity distributions. These discrepancies require new coregistration methods, however most of the existing specialized approaches are either insufficiently automated (Galaxy Community 2022), or closed-source (Abdelmoula *et al.* 2014). We propose a landmark-free streamlined open-source coregistration framework MSIreg, which specifically addresses the characteristics of MSI and H&E data. We demonstrate the versatility and the accuracy of the framework on six Case Studies performed on diverse biological samples and mass spectrometers. The Case Studies include coregistration of single contiguous tissues with large deformations, and simultaneous coregistration of 29 tissue microarray (TMA) cores, with MSI files of up to 16 GB.

## 2 Background

Most existing coregistration methods, such as the one in Galaxy (Galaxy Community 2022), need manual image overlay and manually selected landmarks. Most are also limited to affine transformations, which do not account for local deformations and require multiple iterations for satisfactory performance. As an alternative, Patterson *et al.* (2018) exploited laser ablation marks introduced by matrix-assisted laser desorption/ionization (MALDI) mass spectrometry as ad-hoc landmarks visible in both MSI and H&E. The authors coregistered the landmarks with *scikit-learn* Python package, however the package is limited to affine transformations.

An unsupervised coregistration has been proposed by Abdelmoula *et al.* (2014). The method first reduces the size and the noise of MSI with peak processing. It then generates a representative MSI image with t-distributed stochastic neighbor embedding (t-SNE), coregisters it to the H&E image with a rigid transformation for a same tissue section, and applies an elastic transformation for adjacent tissue sections with the Python *elastix* package (Klein *et al.* 2010). Unfortunately, the method requires different preprocessing for different MS instruments and molecular class. Its implementation is closed-source and requires proprietary software such as FlexImaging (Bruker Daltonics). As another example of unsupervised coregistration, GUI-based M^2^aia (Cordes *et al.* 2021) coregisters a single featured MSI image with the H&E image using the *elastix* package. The single ion is user selected, and it is generally unlikely to contain all the morphological features.

More recently, Race *et al.* (2021) proposed a modified version of the workflow above to coregister and then transfer annotations from H&E images to MSI modality. First, the MSI dataset is reduced in dimensionality to a single representative MSI. Next, a neural network segments an H&E image into regions of differing morphology. Finally, the Python package *elastix* coregisters the representative MSI image and the segmented H&E image. With this approach, the H&E image is reduced to the most prominent tissue structures, which loses local spatial information. The limitations of all the methods above reduce the scope of their practical application.

## 3 Materials and methods

**Figure 1.**
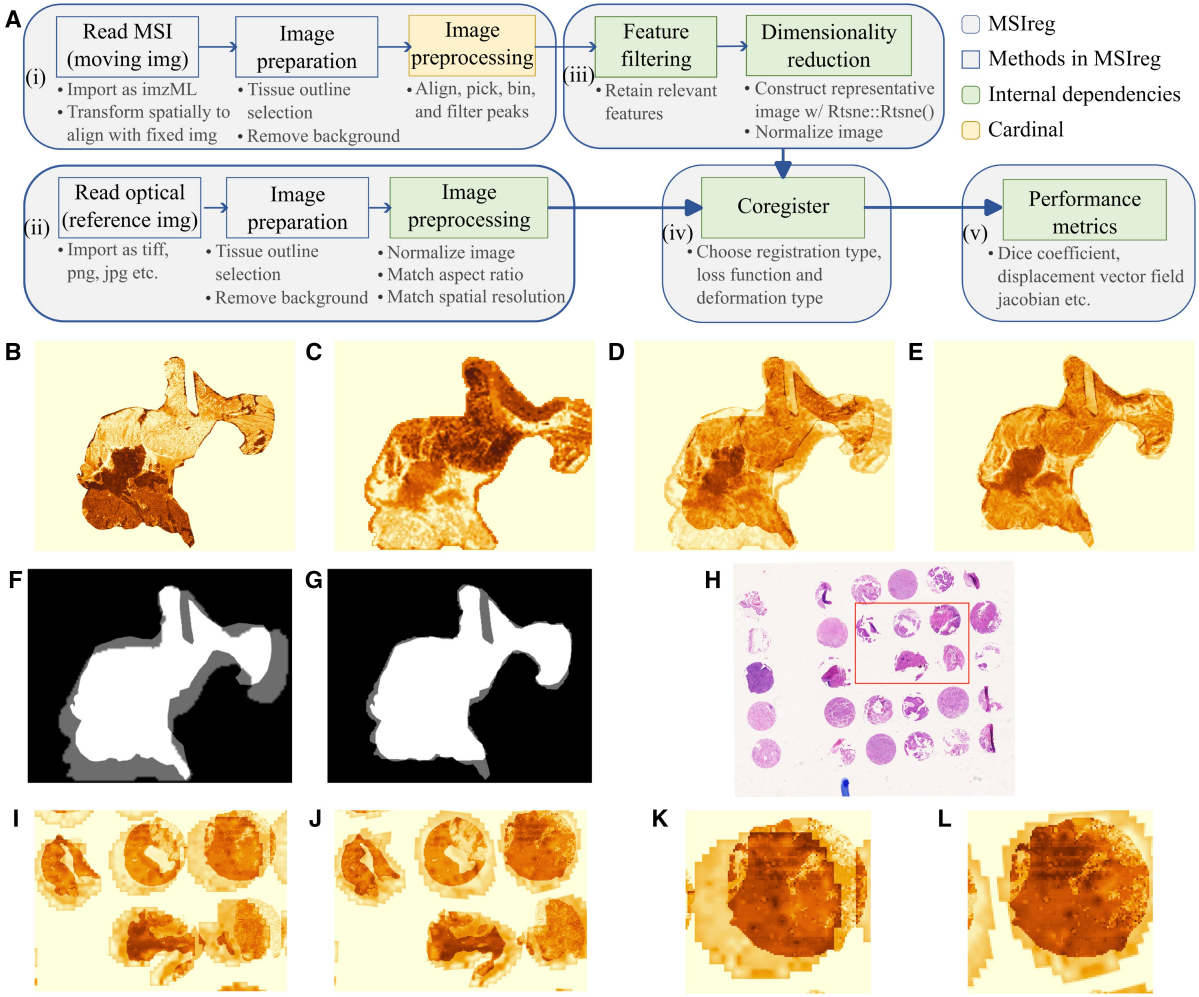
(A) Overview of the MSIreg framework. (B–G) Case Study 1, tissue with elastic deformations. (B) Representative H&E image, (C) representative MS image, (D) overlay of the image pair before coregistration, (E) overlay of the image pair after coregistration, (F) overlay of the tissue masks before coregistration, and (G) overlay of the tissue masks after coregistration. (H–L) Case Study 2, TMA cores with dilated outlines. (H) H&E image before coregistration with a region of interest highlighted (rectangular area), (I) overlay of the image pair of the highlighted region before coregistration, (J) overlay of the image pair of the highlighted region after coregistration, (K) overlay of one tissue core before coregistration, and (L) overlay of one tissue core after coregistration.

We introduce MSIreg, an open-source implementation for coregistration for MS and H&E images. Unlike most existing methods, MSIreg does not require landmarks. Unlike Patterson *et al.* (2018), there is no dependence on the ionization source and is therefore compatible with MS images from any mass spectrometer. MSIreg extends the approach of Abdelmoula *et al.* (2014) in four important directions. First, it standardizes image preparation and processing steps for both modalities, experimental designs, mass spectrometry instruments, and ionization types. Second, it automates filtering of MSI features to improve the signal-to-noise ratio of MS images. Third, MSIreg implements metrics quantifying the quality of coregistration at both global and local scales. Finally, unlike most of the existing state-of-the-art approaches, the implementation is largely automated, requires minimal user input, and is open-source. MSIreg depends on Cardinal for MSI preprocessing, EBImage for H&E image preparation, and SimpleITK for the final registration and and transformation. Figure 1A provides overview of the MSIreg framework.

### 3.1 Preparation and processing of the images

Preparation and processing of MSI and H&E images [Fig. 1A, boxes (i) and (ii)] includes reading in the images, outlining the tissues and removing image background. While MSIreg expects tissue outlines as input, it also includes a functionality to select tissue outline during runtime. Since the images often differ in positions in the frame, amount of background padding, tissue sizes on slide and aspect ratios, image preparation tools also include functionalities that match the aspect ratio, ensure similar background sizes with scaling, cropping and/or padding.

### 3.2 Spatial resolution matching

The spatial resolution of MS images is typically lower than that of H&E images. Therefore, MSIreg uses an upscaling-downscaling strategy to match the spatial resolutions of two images [Fig. 1A, box (ii)]. The MS image is upscaled to a higher resolution, i.e. a factor of its original resolution (spatial_scaling), and the microscopy image is downscaled to match that resolution, which can be user-modified. We found that a spatial_scaling factor of four is generally sufficient.

### 3.3 Feature filtering and dimensionality reduction

An MS image feature represents the abundance of the underlying analyte at various tissue locations. Many analytes are noisy, low-abundant, or localized in a small subregion of the tissue. MSIreg applies an automated selection of spatially informative MSI features [Fig. 1A, box (iii)] using spatial shrunken centroids (Bemis *et al.* 2016). The method combines tissue segmentation with statistical regularization, and outputs a subset of features associated with the segments. Finally, t-SNE summarizes the selected MS image features in a 1D image comparable with the H&E image.

### 3.4 Coregistration

MSIreg leverages the spatial correspondence between the low dimensional embedding of MS and H&E images, and transforms the coregistration problem into an optimization problem [Fig. 1A, box (iv)]. Specifically, MSIreg optimizes mutual information between intensity distributions of the image pair, which removes the need for specifying landmarks prior to coregistration. The optimization is performed by the SimpleITK package with options to select deformation type, optimization type, loss metric and, image sampling strategies. MSIreg also includes utilities to export the coregistered images and transfer annotations from one modality to another.

### 3.5 Performance metrics

MSIreg facilitates both qualitative and quantitative evaluation of coregistration. Qualitative evaluation is subjective, and involves visual overlays described in Section 4. Implementing quantitative metrics is more challenging. Previously proposed metrics such as dice coefficient and Euclidean distance for pixels with common morphology (Abdelmoula *et al.* 2014) require the user to annotate the representative locations in both images. The process resembles manual landmarking, and is not compatible with an automated workflow. To address this, MSIreg implements quantitative metrics that only rely on tissue margin masks and on transformation parameters of the coregistration. MSIreg quantifies global accuracy of coregistration by applying dice coefficient to tissue masks, and by calculating normalized cross correlations of representative images’ intensities. MSIreg quantifies local (and elastic) accuracy of coregistration using the displacement field Jacobian determinant (itk.org/Doxygen/html/classitk_1_1DisplacementFieldJacobianDeterminantFilter.html). At every tissue location, the Jacobian determinant quantifies the extent to which the moving image expanded, contracted, or folded. Detailed descriptions of the metrics are provided in Vignette 1.

### 3.6 Implementation and documentation

Details of package use, as well as the Case Studies, are available in the package Vignettes. The package is available and tested on MacOS, Linux. It is not available on Windows directly owing to the unavailability of an R wrapper for SimpleITK. However, a workaround for Windows OS using Windows subsystem for Linux (WSL) has been established. This setup is described in the package Vignette 2. The package was tested on files of up to 26 GB. The Case Studies in the Vignettes were performed on an Apple M2 Max MacBook Pro with 10 core CPU and 32 GB memory, each completing in under 50 min.

## 4 Case studies

We evaluated MSIreg on six experimental Case Studies that represent experiments with different MSI ionization sources, spatial and mass resolutions, extent of spatial deformation, and tissue types. Package Vignette 1 describes the performance metrics, and Vignette 2 describes the installation instructions and conversion tools from RAW files to imzML files. Vignette 3 (Case Study 1) showcases the ability of MSIreg to coregister high mass spectral resolution images and large spatial deformations. Vignette 4 (Case Study 2) illustrates simultaneous coregistration of multiple TMA cores. Vignettes 5–8 contain additional Case Studies that illustrate the ability of MSIreg to handle a combination of varying spatial resolution and noise of MS images. Finally, Vignette 9 contains an end to end downstream analysis of tumor-stroma classification on MS images after coregistration. All Case Studies were performed with default coregistration parameters (type: bspline, optimizer: textttlbfgsb, metric: mattesMI, interpolator: linear). We summarize the first two Case Studies below.

### 4.1 Case study 1: tissue with elastic deformations

#### 4.1.1 Experimental details

The MS image of a tissue with colorectal cancer (Moritz *et al.* 2023) was acquired with 4800 MALDI-TOF/TOF Analyzer (Applied Biosystems, Waltham, MA, USA), with a step size of 150 µm, and mass range of 800–2500 *m*/*z*. Following MS image acquisition, the H&E stained tissues were scanned at x20 magnification with the Axio Scan Z1 (ZEISS, Göttingen, Germany).

#### 4.1.2 Coregistration challenges

The Case Study had two challenges. First, the two images had a substantial discrepancy in the selected tissue outline (Fig. 1B and C). For example, the H&E image excluded the adipose fat region (middle right), however this region was present in MSI. Second, the MSI and H&E images had large and elastic deformations inside the tissues (Fig. 1D and F). Such discrepancies severely undermine the downstream interpretation.

#### 4.1.3 Coregistration results

MSIreg rectified the discrepancies between the images, and improved alignment of tissue morphology (Fig. 1E). The tissue masks (Fig. 1F and G) show that the method successfully adapted to the inconsistencies between the tissue outlines. In particular, MSIreg did not contract the MS image to match the H&E. The dice coefficient improved from 82.88% before coregistration to 95.20% postcoregistration. The intensity correlation improved from 0.47 to 0.6. Visual analysis of the displacement field Jacobian determinant is available in Vignette 3.

### 4.2 Case study 2: TMA cores with dilated outlines

#### 4.2.1 Experimental details

The MS image of 29 tissues with urothelial cancer (Föll *et al.* 2022) was acquired with 4800 MALDI-TOF/TOF Analyzer, using the 4000 Series Explorer for instrumentation calibration. The raster step size of the image was 150 µm, resulting in 500 laser shots per spectrum. The mass spectra were captured in positive ion reflection mode with a mass range between 800 and 2300 *m*/*z*. To acquire the H&E image, hemalum staining was performed after matrix removal and tissues scanned using Axio Scan Z1 at x20 magnification (ZEISS, Göttingen, Germany).

#### 4.2.2 Coregistration challenges

The Case Study presents the challenge of a simultaneous coregistration of 29 tissue microarray (TMA) cores. The small cores moved on the slide during the staining procedure, causing independent misalignment between MS and H&E images of each core. To create an additional challenge, we deliberately padded the background beyond the tissue outline of the H&E image. Such situations commonly arise when imaging the whole slide, including the background. Figure 1I and K show large translatory and rotatory deformations.

#### 4.2.3 Coregistration results

MSIreg successfully aligned the cores in MS image with those in H&E image but not the padding. This illustrates the importance of coregistering the intensity distributions of the images, as opposed to the outlines (Fig. 1J–L). The dice coefficient improved from 48.1% to 86.5% post coregistration while the intensity correlation improved from 0.39 to 0.67. Detailed analysis for evaluating the local coregistration performance and the effects of dilated tissue outline is presented in Vignette 4.

## 5 Discussion

MSIreg implements open-source unsupervised coregistration between MS and H&E images with minimal user input. The landmark-free workflow relies on tissue heterogeneity to distinguish tissue sub-regions, minimizes user input and is largely automated. In absence of heterogeneous morphology, the tissues are effectively coregistered through their outlines. We believe that MSIreg is a useful step toward joint interpretation of these images in clinical applications.

## Data Availability

The data analyzed in this article are derived from PRIDE, with project IDs PXD039409 (https://www.ebi.ac.uk/pride/archive/projects/PXD039409) and PXD026459 (https://ebi.ac.uk/pride/archive/projects/PXD026459).
